# Revisiting Jenkins’ Rule: Evidence-Based Insights on the Suture-to-Wound Length Ratio and Wound Dehiscence

**DOI:** 10.7759/cureus.98942

**Published:** 2025-12-10

**Authors:** Jideofor Okoye, Ammar M Eskander, Kiranjot Kaur, Beshr Mosa Basha, Osasenaga Bencharles, Shashwat Shetty, Noman Ansari

**Affiliations:** 1 Trauma and Orthopaedics, Mersey and West Lancashire Teaching Hospitals NHS Foundation Trust, England, GBR; 2 General Surgery, Dr. Sulaiman Al-Habib Hospital, Al Khobar Branch, Al Khobar, SAU; 3 US Navy, United States Military, Great Lakes, USA; 4 Clinical Research, Arizona State University, Tempa, USA; 5 Medicine, Shri B. M. Patil Medical College, Bijapur, IND; 6 General Surgery, Dr. Sulaiman Al-Habib Hospital, Al Khobar, SAU; 7 Medicine and Surgery, University Hospitals of Derby and Burton NHS Foundation Trust, Derby, GBR; 8 Orthopaedics, Hillingdon Hospital, Uxbridge, GBR; 9 General Medicine, Nishtar Medical University Hospital, Karachi, PAK

**Keywords:** abdominal closure, jenkins’ rule, short-stitch technique, suture-length to wound-length ratio, wound dehiscence

## Abstract

Wound dehiscence is a serious postoperative complication, with fascial closure technique and suture-length to wound-length (SL:WL) ratio being key determinants of wound integrity. Jenkins’ rule, recommending a 4:1 SL:WL ratio, has long guided midline laparotomy closure. Recent evidence suggests that optimal ratios should be tailored to the surgical context, patient factors, and suture material. High-tension closures, such as emergency laparotomies in obese patients, benefit from higher ratios of 5:1-6:1, while elective or low-tension procedures, including laparoscopic port-site closures, may achieve secure outcomes with ratios of 3.5:1-4:1. Modern suture materials, including monofilament, delayed-absorbable, and barbed sutures are combined with fine-bite or short-stitch techniques, enhance fascial approximation, reduce tissue trauma, and allow flexible ratio application. This systematic review of five studies with a combined sample size of 1,431 patients confirms that Jenkins’ 4:1 rule remains a reliable baseline. However, SL:WL ratios should be adapted to wound tension, surgical setting, and patient characteristics. Tailoring suture length to these factors can optimize fascial closure, minimize wound dehiscence, and improve postoperative outcomes, providing a modern, evidence-based refinement of Jenkins’ foundational principle.

## Introduction and background

Surgical wound dehiscence remains one of the most serious postoperative complications, contributing significantly to patient morbidity, prolonged hospitalization, and increased healthcare costs. Among the technical factors influencing dehiscence, the method of fascial closure and the ratio of suture length to wound length (SL:WL) have consistently been identified as critical determinants of wound integrity [1]. In 1976, Jenkins introduced a mathematical principle for midline laparotomy closure, demonstrating that when the suture length was at least four times the wound length, referred to as a 4:1 ratio, the risk of dehiscence was markedly reduced [2]. This concept, now widely known as Jenkins’ rule, has since become a cornerstone of safe and effective fascial closure techniques [3]. The principle is based on achieving even tension distribution across the wound and accommodating normal postoperative tissue expansion without compromising perfusion. Over the past few decades, several developments have challenged the universality of this rule. The introduction of modern suture materials such as monofilament delayed-absorbable sutures, barbed sutures, and fine continuous closure techniques has improved tensile strength, handling, and biocompatibility while reducing tissue trauma [4].

At the same time, surgical practice and patient demographics have evolved. Rising rates of obesity, emergency procedures, and minimally invasive surgeries have altered the biomechanical conditions of wound healing. These changes raise a crucial question: Does the traditional 4:1 ratio still represent the optimal balance between tension relief and tissue integrity in contemporary surgical practice? Recent research indicates that the 4:1 ratio remains a reliable baseline. However, higher ratios (5:1-6:1) may further reduce wound dehiscence in high-tension closures, such as in obese or re-operative abdominal wall patients [5]. Conversely, in low-tension settings, such as laparoscopic port-site closures, slightly lower ratios (around 3.5:1 to 4:1) may provide adequate strength without increasing the risk of wound failure. These findings suggest that Jenkins’ rule should not be regarded as a fixed standard but rather as a flexible guideline that can be adapted to the surgical context, suture material, and tissue tension. Consequently, there is a growing need to revisit and refine Jenkins’ rule in light of current surgical techniques and material advancements, aiming to establish an evidence-based, context-specific framework for optimal wound closure.

The primary aim of this study is to systematically evaluate contemporary evidence (2010-2024) on the relevance and validity of Jenkins’ rule, determining whether the 4:1 suture-length to wound-length (SL:WL) ratio remains optimal for minimizing wound dehiscence across different surgical settings. The secondary aim is to assess how factors such as suture material, closure technique, anatomical site, and patient characteristics influence the relationship between the SL:WL ratio and the risk of wound dehiscence, and to propose an updated, evidence-based perspective for modern surgical practice.

## Review

Materials and methods

Search Strategy

This systematic review was conducted in accordance with the Preferred Reporting Items for Systematic Reviews and Meta-Analyses (PRISMA) 2020 guidelines to ensure methodological transparency and reproducibility [6]. A comprehensive electronic search was performed across PubMed, Embase, Scopus, and the Cochrane Library to identify relevant studies published between January 2010 and December 2024. The search combined controlled vocabulary (MeSH/Emtree terms) and free-text keywords including “Jenkins’ rule,” “suture length to wound length ratio,” “fascial closure,” “abdominal wound dehiscence,” and “suture technique.” Boolean operators (“AND,” “OR”) were applied to refine results. Reference lists of included studies and key reviews were manually screened to identify additional eligible publications. Search results were imported into EndNote for organization and duplicate removal.

Eligibility Criteria

Eligibility was defined using the PICO framework to ensure methodological rigor [7]. The population included adult patients undergoing abdominal or fascial wound closure. The intervention involved closure following Jenkins’ rule or studies measuring the SL:WL ratio. Comparators included alternative closure techniques or materials not adhering to Jenkins’ rule. The primary outcome was wound dehiscence, with secondary outcomes including related closure complications. Eligible designs were randomized controlled trials (RCTs), cohort, and comparative studies, while animal studies, case reports, editorials, non-English articles, and abstracts were excluded.

Study Selection

All identified records were imported into a citation manager, and duplicate entries were automatically removed before screening. Two reviewers independently screened titles and abstracts to exclude irrelevant studies, following the PRISMA 2020 flow protocol. Full-text versions of potentially eligible articles were retrieved for in-depth evaluation against the inclusion criteria. Any disagreements regarding study inclusion were resolved by consensus or consultation with a third reviewer. The entire selection process, including the number of records at each stage, is presented in a PRISMA flow diagram to ensure methodological transparency and reproducibility.

Data Extraction

Data were extracted using a standardized template that included study characteristics, population demographics, type of surgical procedure, closure technique, suture material, SL:WL ratio, anatomical site, dehiscence rate, and follow-up duration. Data extraction was independently verified by two reviewers to minimize transcription errors and ensure data reliability. Due to heterogeneity in surgical procedures, patient populations, and SL:WL ratios, a meta-analysis was not performed. A narrative synthesis was conducted instead.

Risk-of-Bias Assessment

The methodological quality of RCTs was evaluated using the RoB 2 (Revised Cochrane Risk-of-Bias Tool) [8], while non-randomized studies were assessed with the ROBINS-I (Risk of Bias in Non-randomized Studies of Interventions) tool [9]. Each study was categorized as having low, moderate, or high risk of bias based on factors such as randomization, confounding, attrition, and reporting bias.

Data Synthesis

A qualitative narrative synthesis was performed due to heterogeneity in study designs, surgical populations, and outcome measures. Data were analyzed to identify consistent trends between SL:WL ratios and wound dehiscence rates across surgical contexts. When comparable data were available, pooled descriptive statistics were considered to highlight effect size directionality. Findings were integrated with methodological assessments to ensure balanced and statistically sound interpretation.

Results

Study Selection Process

**Figure 1:**
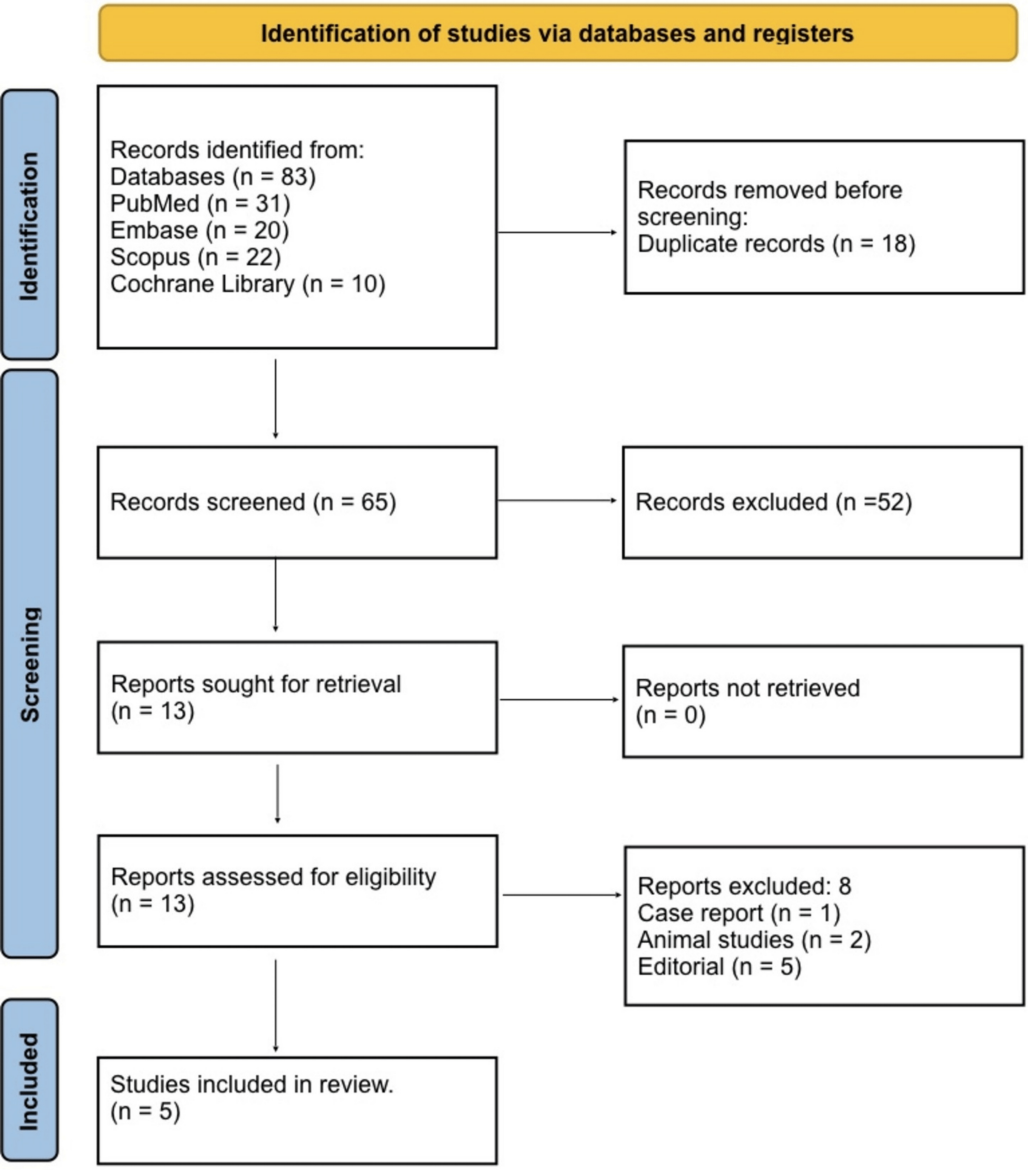
PRISMA 2020 flow diagram. PRISMA, Preferred Reporting Items for Systematic Reviews and Meta-Analyses

A total of 83 records were identified through database searching, including 31 from PubMed, 20 from Embase, 22 from Scopus, and 10 from the Cochrane Library (Figure 1). After the removal of 18 duplicate records, 65 unique studies remained for title and abstract screening. Following this initial screening, 52 studies were excluded as they did not meet the inclusion criteria based on relevance or study design. The full texts of 13 articles were retrieved and assessed for eligibility. Of these, eight reports were excluded, comprising case reports, animal studies, editorials, and conference abstracts, leaving a total of five studies that met all inclusion criteria and were included in the final qualitative synthesis.

Characteristics of the Selected Studies

Table 1 summarizes key studies evaluating the SL:WL ratio and its impact on wound dehiscence. Millbourn et al. reported that short-stitch closures with SL:WL ≥4:1 in 737 midline laparotomies reduced hernia rates (5.6% vs. 18%) compared to long-stitch closures [10]. In 100 abdominal closures, Williams et al. observed that achieving SL:WL ≥4:1 lowered wound complications, while suboptimal ratios increased infection and hernia risk [11]. In the STITCH trial, Deerenberg et al. found that small-bite closures (~5:1 SL:WL) significantly reduced hernia incidence (13% vs. 21%) compared to large-bite closures (~4.3:1) [12]. A 2021 educational intervention [13] improved SL:WL compliance from 76% to 90%, highlighting the role of training. Frassini et al. recommended an SL:WL ratio of ≥4:1 for emergency midline laparotomies, demonstrating that higher ratios reduce wound complications in high-tension settings [14]. Across studies, the midline fascia was the primary anatomical site, continuous closure was commonly used, and monofilament sutures (polydioxanone (PDS)) were typical. SL:WL ratios of 4:1-5:1 were generally associated with optimal wound integrity and lower dehiscence rates.

**Table 1:** Characteristics of the selected studies. SL:WL, suture-length to wound-length; RCT, randomized controlled trial; PDS, polydioxanone

Authors and year	Population (P)	Exposure/Condition (I)	Comparator (C)	Outcomes (O) - wound dehiscence/incisional hernia	Pathophysiological findings	Anatomical impact	Dominant suture type	Closure technique	Typical SL:WL ratio	Dehiscence/hernia rate (%)	Key findings
Millbourn et al. (2009) [10]	Adults undergoing midline incisions (n = 737)	Short‑stitch group (<10 mm bites)	Long‑stitch group (≥10 mm bites)	Wound dehiscence: 1/381; hernia: long 18% vs. short 5.6%	Short‑stitch and higher SL:WL may improve fascial strength	Midline laparotomy fascia	Not specified	Single-layer continuous running	At least 4:1	Hernia: 18% vs. 5.6%	Supports >4:1 ratio and short-stitch to reduce hernia incidence
Williams et al. (2017) [11]	100 consecutive abdominal wall closures by residents	Measurement of SL:WL achieved	Not mentioned	SL:WL ≥4:1 achieved in 76%; higher infection/hernia when <4:1	Lower SL:WL associated with worse wound outcomes	Abdominal wall fascial closure	Not specified	Continuous fascia closure	Mean ~4.6:1	Not fully specified	Highlights that real-world compliance with the 4:1 rule is suboptimal
Deerenberg et al. (2015) [12]	Elective midline laparotomy, n=560	Small‑bite closure (5 mm from edge, 5 mm apart)	Large‑bite closure (10 mm/10 mm)	Incisional hernia: small 13% vs. large 21% at 1 year	Small‑bite plus higher SL:WL distributes tension better	Midline abdominal wall	Monofilament PDS	Continuous small‑bite vs. large‑bite	Small ~5:1; large ~4.3:1	Hernia: 13% vs. 21%	Small-bite technique with higher SL:WL improves hernia outcomes
Seth et al. (2021) [13]	Midline laparotomy closures pre- and post-education (n=200)	Education to achieve SL:WL ≥4:1	Pre-education group	SL:WL ≥4:1 achieved 76% pre vs. 90% post	Education improves compliance with SL:WL and outcomes	Midline fascia	Not specified	Continuous closure	Target ≥4:1	Not mentioned	Education improves the ratio of attainment and closure outcomes
Frassini et al. (2023) [14]	Emergency midline laparotomy	Recommendation: SL:WL ≥4:1	Standard closure (<4:1)	SL:WL ≥4:1 reduces incisional hernia/wound complications	A high ratio helps distribute tension in emergency closures	Midline abdominal wall	Not specified	Continuous midline closure	≥4:1	Not mentioned	Guideline-level support for ≥4:1 ratio in emergencies

Risk-of-Bias Assessment

The risk of bias across the selected studies varied according to design and methodology. Millbourn et al. conducted a prospective cohort study with a large sample, but the lack of randomization, potential confounding, and absence of blinding resulted in a moderate risk of bias [10]. Similarly, Williams et al. performed a prospective observational study with a small sample, operator variability, and no randomization, leading to a moderate risk [11]. In contrast, the STITCH trial by Deerenberg et al., a multicenter RCT with proper randomization, outcome assessor blinding, and low attrition, was rated as low risk [12]. Seth et al. implemented a single-center prospective educational intervention, which was non-randomized and subject to selection bias, resulting in moderate risk [13]. Finally, Frassini et al. reported a guideline-based observational study in an emergency setting with heterogeneous procedures and potential confounding, also rated moderate risk [14], as shown in Table 2.

**Table 2:** Risk-of-bias assessment. RCT, randomized controlled trial; ROBINS-I, Risk of Bias in Non-randomized Studies of Interventions; RoB 2, Risk of Bias 2 tool

Study	Study design	Tool	Risk rating	Justification
Millbourn et al. (2009) [10]	Prospective cohort	ROBINS-I	Moderate	Large sample, but non-randomized and potential confounding; blinding not performed
Williams et al. (2017) [11]	Prospective observational	ROBINS-I	Moderate	Small sample; performed by trainees, potential for operator bias; no randomization
Deerenberg et al. (2015) [12]	Randomized controlled trial (RCT) (STITCH)	RoB 2	Low	Multicenter RCT; randomization, blinding of outcome assessors, low attrition
Seth et al. (2021) [13]	Prospective interventional	ROBINS-I	Moderate	Single-center educational intervention; non-randomized; potential selection bias
Frassini et al. (2023) [14]	Guideline-based observational	ROBINS-I	Moderate	Observational in an emergency setting; heterogeneity in procedures; potential confounding

Discussion

The findings of this systematic review reaffirm the central role of the SL:WL ratio in minimizing wound dehiscence, consistent with Jenkins’ original 4:1 recommendation. Millbourn et al.demonstrated that midline laparotomies closed with short-stitch techniques, achieving SL:WL ≥4:1, had lower hernia rates (5.6% vs. 18%), emphasizing the importance of adequate suture length for tension distribution [10]. Williams et al. similarly found that maintaining SL:WL ≥4:1 reduced wound complications and infections, while suboptimal ratios were associated with higher risk, highlighting real-world challenges in achieving the recommended ratio [11]. The STITCH trial by Deerenberg et al. further confirmed that small-bite closures with SL:WL ratios around 5:1 significantly reduced incisional hernia rates (13% vs. 21%) compared to large-bite closures, supporting the superiority of higher ratios and fine-bite techniques in elective midline laparotomies [12].

Educational interventions by Seth et al. demonstrated that targeted training improved compliance with the 4:1 rule from 76% to 90%, indicating that surgeon education is crucial in optimizing fascial closure outcomes [13]. Frassini et al. recommended SL:WL ≥4:1 in emergency midline laparotomies, showing that higher ratios effectively reduce wound complications in high-tension settings [14]. These studies collectively support a stratified approach to fascial closure: elective or moderate-tension closures can reliably follow the classic 4:1 ratio, optimized short-stitch techniques with SL:WL ~5:1 reduce dehiscence in elective procedures, and emergency or high-tension closures benefit from ratios ≥5:1. Modern suture materials, including monofilament and delayed-absorbable types, facilitate uniform tension distribution, allowing flexibility in SL:WL ratios while minimizing tissue trauma.

Several limitations must be acknowledged. The included studies were heterogeneous in terms of patient populations, surgical settings, closure techniques, and suture materials, limiting direct comparability. Some studies had small sample sizes or single-center designs, increasing the risk of bias and reducing external validity [10,11]. Additionally, follow-up durations were often short, potentially underestimating late dehiscence or hernia formation, and most studies focused on midline laparotomies, leaving other anatomical sites underrepresented. Future research should focus on large, multicenter trials stratified by patient body mass index (BMI), comorbidities, and surgical urgency to validate optimal SL:WL ratios across diverse populations. Biomechanical and imaging studies assessing fascial tension and perfusion could refine closure techniques, while investigations into novel suture materials, including barbed or hybrid absorbable sutures, may further improve outcomes. Development of procedure-specific, evidence-based SL:WL guidelines and long-term follow-up studies evaluating hernia formation, chronic pain, and wound integrity are needed to optimize modern fascial closure practices.

## Conclusions

Jenkins’ 4:1 SL:WL ratio remains a reliable baseline, but modern evidence supports adjusting the ratio based on wound tension, patient factors, and suture type. High-tension closures may benefit from ratios of 5:1-6:1, while low-tension closures may use 3.5:1-4:1. Flexible application of Jenkins’ rule can optimize wound integrity and reduce the risk of dehiscence in modern surgical practice.
